# Diet and Environment Shape Fecal Bacterial Microbiota Composition and Enteric Pathogen Load of Grizzly Bears

**DOI:** 10.1371/journal.pone.0027905

**Published:** 2011-12-15

**Authors:** Clarissa Schwab, Bogdan Cristescu, Joseph M. Northrup, Gordon B. Stenhouse, Michael Gänzle

**Affiliations:** 1 Department of Agricultural, Food and Nutritional Science, University of Alberta, Edmonton, Alberta, Canada; 2 Department of Biological Sciences, University of Alberta, Edmonton, Alberta, Canada; 3 Foothills Research Institute, Hinton, Alberta, Canada; J. Craig Venter Institute, United States of America

## Abstract

**Background:**

Diet and environment impact the composition of mammalian intestinal microbiota; dietary or health disturbances trigger alterations in intestinal microbiota composition and render the host susceptible to enteric pathogens. To date no long term monitoring data exist on the fecal microbiota and pathogen load of carnivores either in natural environments or in captivity. This study investigates fecal microbiota composition and the presence of pathogenic *Escherichia coli* and toxigenic clostridia in wild and captive grizzly bears (*Ursus arctos*) and relates these to food resources consumed by bears.

**Methodology/Principal Findings:**

Feces were obtained from animals of two wild populations and from two captive animals during an active bear season. Wild animals consumed a diverse diet composed of plant material, animal prey and insects. Captive animals were fed a regular granulated diet with a supplement of fruits and vegetables. Bacterial populations were analyzed using quantitative PCR. Fecal microbiota composition fluctuated in wild and in captive animals. The abundance of *Clostridium* clusters I and XI, and of *C. perfringens* correlated to regular diet protein intake. Enteroaggregative *E. coli* were consistently present in all populations. The *C. sordellii* phospholipase C was identified in three samples of wild animals and for the first time in *Ursids*.

**Conclusion:**

This is the first longitudinal study monitoring the fecal microbiota of wild carnivores and comparing it to that of captive individuals of the same species. Location and diet affected fecal bacterial populations as well as the presence of enteric pathogens.

## Introduction

The gastrointestinal tract of mammals is a complex ecosystem resulting from a dynamic interplay between diet, host, and commensal bacteria. The composition of the intestinal microbiota depends on physiology of the gut as well as the type of diet (herbivorous-omnivorous-carnivorous) [1].

*Ursids* including grizzly bears (*Ursus arctos*) are fast digesters with a simple digestive tract composed of stomach, short small intestine, indistinct hindgut, and no cecum [2], [3]. The diet of grizzly bears varies with season and local food availability [4]. Throughout their range, grizzly bears feed on plants (roots, forbs, grasses, berries), plant concentrates such as seeds, and animal protein, with changing proportions from spring to fall [5]. The herbivorous giant panda (*Ailuropoda melanoleuca*) and grizzly bears of interior wild populations consume a predominantly vegetative diet. Their fecal bacterial populations are characterized by a predominance of the facultative anaerobes *Enterobacteriacae* and enterococci [6], [7]. The presence of short chain fatty acids (SCFAs) in grizzly bear feces indicates intestinal metabolic activity and contribution of the gut microbiota to energy maintenance [6].

In domesticated animals, diet alterations affect intestinal microbiota composition, host resistance and susceptibility to potentially harmful bacterial pathogens, such as pathogenic *Escherichia coli* (enterotoxinogenic *E. coli* (ETEC), enteropathogenic *E. coli* (EPEC), or shiga-toxin producing *E. coli*), or *Clostridium* spp. [8]. Although *Enterobacteriaceae* and clostridia are predominant bacterial groups in the intestine of grizzly bears [6], no data exist on the presence of enteric pathogens in these animals.

Free ranging wild animals from small populations of conservation concern may be particularly vulnerable to habitat, dietary and pathogenic stressors [9]. Management plans for such populations would benefit from finer scale understanding of the relationship between food availability and gut physiology as well as pathogen load information. Also, such information can be useful in designing nutritional programs for captive bears preventing pathogen spread. The grizzly bear was recently designated as Threatened in Alberta (Canada) in response to low numbers revealed by DNA-based population estimates for the province [10]. Little is known of the bacterial pathogens associated with grizzly bears and even less is known of the relation between habitat and physiology of the gut.

To investigate how environmental factors affect the intestinal microbiota of the grizzly bear, we surveyed a total of 10 adult grizzly bears, of which 2 were housed in captivity, 4 were wild bears from population 1, and 4 bears from population 2. Captive bears were monitored for 6 months, whereas wild bears were monitored for 4 months (population 1) and 6 months respectively (population 2), with the shorter monitoring period for population 1 due to GPS radiocollar malfunction or premature collar removal by the by the bears themselves.

Since intestinal microbiota are unique to each individual, the longitudinal comparison within the same animal enables determination of population variability and detection of changes induced by shifts in diet or disease [11]–[13]. Composition of the fecal microbiota was determined by group specific quantitative PCR targeting nine bacterial groups highly abundant in mammals. The presence of pathogenic *E. coli* and *Clostridium* spp. was analyzed by detection of virulence and toxin genes. Food items in the diet were determined visually.

## Results

### Diet content of wild and captive grizzly bears

**Figure 1:**
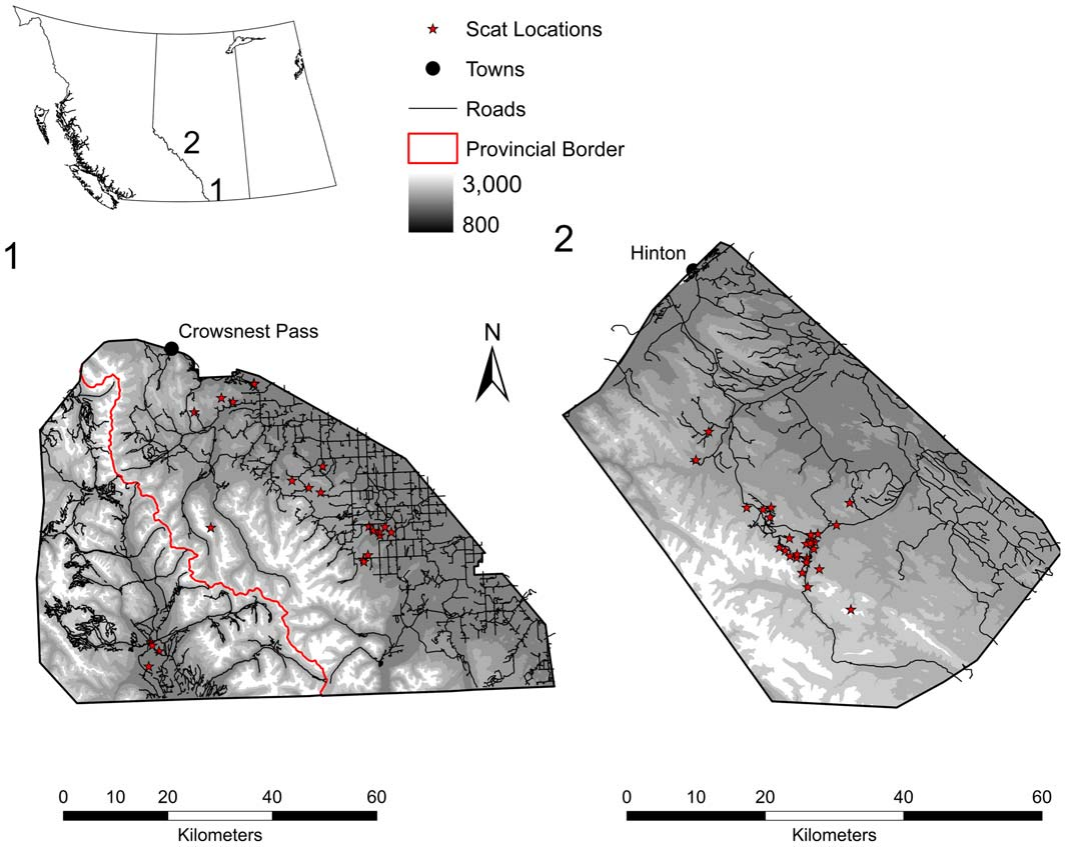
Sampling areas for wild grizzly bear populations in Alberta, Canada.

The diet of wild grizzly bears was diverse, and varied among individuals in each population, as well as between populations (Figure S1). Plant material was the major contributor to the diet of animals in both populations (sample location and information on individual bears are provided in Figure 1 and Table 1). Bears from population 1, inhabiting an agriculturally dominated landscape, contained a higher proportion of cereals in their diet. Mammalian matter was present in 3 out of 5, and 8 out of 16 analyzed samples of grizzly bears W1 and W2 of population 1, respectively. In population 2, 5 of 11 and 3 of 12 feces of grizzly bears W6 and W5, respectively, contained mammalian matter. These results relate to the availability of food items in the two habitats as well as the choices of individual bears. Agricultural lands in south-western Alberta are used for cattle grazing and dead cattle are available for bears to scavenge on throughout the year. In west-central Alberta, domesticated animals are not available and bears must hunt or opportunistically scavenge wild ungulates. Captive grizzly bears obtained a regular diet containing 24–31% protein, 15–18% fat, and 0.37% fibre.

**Table 1:** Animals monitored in this study.

Population	Location	Animal	Sex	Age in years (in 2009)	Animal ID
1	South-western Alberta	G077	F	8	W1
		G090	M	Approx. 10	W2
					
		G084	M	6	W3
		G125	M	nd^b^	W4
2	West-central Alberta	G023^a^	F	20	W5
		G113	F	7	W6
		G112	M	Approx. 2	W7
		G115	M	nd	W8
3	Calgary Zoo	ZF	F	19	C1
		ZM	M	22	C2

a first fecal sample taken at capture, not included in visual analysis of fecal dietary content,

b not determined.

### Composition of wild and captive grizzly bear fecal microbiota

**Figure 2:**
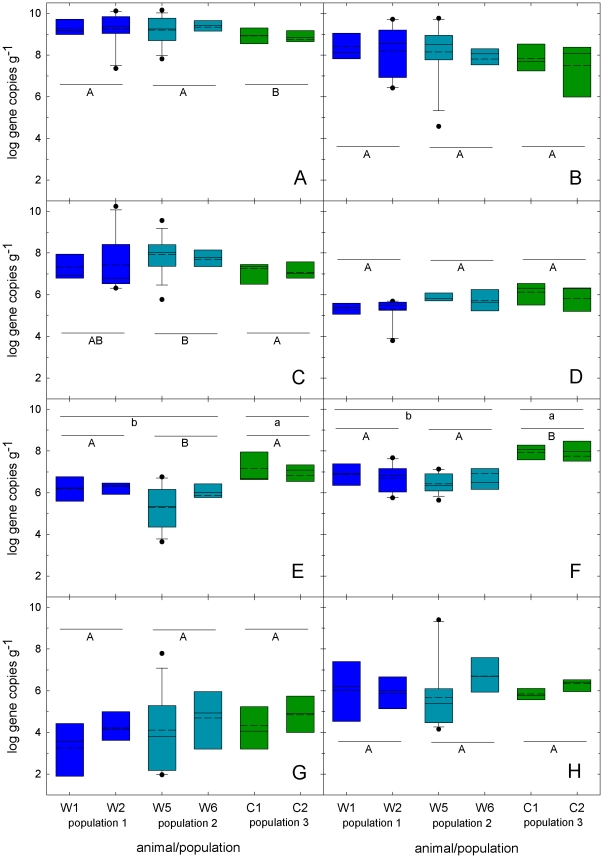
Bacterial groups present in the feces of wild and captive grizzly bears. Bacterial groups (A, total Eubacteria; B, *Enterobacteriaceae*; C, *Enterococcus*; D, *Lactobacillus*, *Pediococcus*, *Leuconostoc*, *Weissella*; E, *Clostridium* cluster I; F, *Clostridium* cluster XI, G, *Clostridium* cluster XIV; H, *Bacteroides-Prevotella-Porphyrmonas*) detected in feces of a four wild (W1,W2, W5, and W6) and two captive (C1, C2) grizzly bears during sampling in 2009. Box plots show the 25 to 75% percentile range of the data in the box, the 5 to 95% range (whiskers), the median (middle line), and the mean (dashed line). Outliers are indicated by dots. Boxplots corresponding to the three populations are indicated by colour (population 1 blue, population two turquois, population 3 green). Differences between populations were analysed using one-way ANOVA. If data was not distributed normally, Kruskal-Wallis One Way Analysis of Variance on Ranks was applied (total Eubacteria, *Enterobacteriaceae*, *Enterococcus*, *Clostridium* cluster XI). Populations that do not share a common superscript (A,B) are significantly different (p<0.05). Abundance of *Clostridium* clusters I and XI was also compared in wild and captive animals (Kruskal-Wallis One Way Analysis of Variance on Ranks); their abundance was significantly different (p<0.05) if values for wild and captive animals do not share a common superscript (a,b).

Bacterial populations in feces of ten grizzly bears from three different populations were determined using quantitative PCR (Table S1 and Figure 2). Total Eubacteria counts were significantly higher in the feces of wild grizzly bears compared to captive bears (p<0.05). Enterococci and *Enterobacteriacae* were prominent bacterial groups in the feces of all bears monitored. In the feces of wild bears, *Bacteroides-Prevotella-Porphyrmonas* and the *Clostridium* clusters I and XI were present in similar numbers, whereas in the feces of captive animals, clostridia of clusters I and XI were significantly higher (p<0.05). The *Clostridium* cluster XIV was detectable in low numbers in the majority of the samples; bifidobacteria and the *Clostridium* cluster IV were present at about log 3 gene copies g^−1^ feces, or below detection limit in all three populations (data not shown).

The abundance of *Clostridium* cluster I and XI were significantly higher in captive than in wild animals (p<0.05, Figure 2). In captive bears, counts of *Clostridium* cluster I and XI highly correlated to total bacterial counts (r = 0.82, p<0.001 and r = 0.70, p<0.001, respectively) and to each other (r = 0.78, p<0.001). In contrast, *Clostridium* clusters I correlated to *Clostridium* clusters XI and XIV (r = 0.71, p<0.05 and r = 0.79, p<0.001, respectively) in population 1 and to the *Bacteroides-Prevotella-Porphyrmonas* group in population 2 (r = 0.74, p<0.001).

### Long-term monitoring of grizzly bear fecal microbiota

For two bears of each population, fecal microbiota were monitored over a period of 2 to 6 months (Figure 2). In wild bears, total bacterial counts increased in May and June as the microbiota of bears adjusted during the transition from spring (after den emergence) to early summer (data not shown). This trend was observed in both study areas. Bacterial populations fluctuated extensively in wild and captive animals; the degree of fluctuations was indicated by the extent of variation in the box plots and was most pronounced in two wild animals W2 and W5. However, in the majority of samples the relative rank of bacterial populations remained constant.

### Enteric pathogen load in feces of wild and captive grizzly bears

Due to the high abundance of *Enterobacteriaceae* and the clostridial clusters I and XI, quantitative PCR was employed to detect virulence factors of pathogenic or toxinogenic species in these taxonomic groups (Table 2). *Clostridium perfringens* (*Clostridium* cluster I) alpha-toxin gene *cpA* was consistently detected in the feces of captive grizzly bears. The gene encoding the phospholipase C *cspC* of *C. sordellii* (*Clostridium* cluster XI) was identified in 3 samples from wild animals. All samples were negative for *C. botulinum* (*Clostridium* cluster I) neurotoxin genes A and B, and *C. difficile* toxin B *tcdB* (*Clostridium* cluster XI). The gene encoding the heat-stable enterotoxin EAST of enteroaggregative *E. coli* (EAEC) and other pathogenic *E. coli* was present in all samples. The genes encoding the heat labile enterotoxin (LT) and the heat stable enterotoxin STa of enterotoxigenic *E. coli* were identified in individual samples but were not consistently present.

**Table 2:** Presence of pathogen toxin genes in feces of wild and captive grizzly bears.

		Clostridial toxin genes [log gene copy numbers g^−1^]		*Enterobacteriacae* toxin genes [log gene copy numbers g^−1^]		
Population	Animal (number of samples)	*C. perfringens* alpha toxin A *cpa*	*C. sordellii* phospholipase C *cspC*	EAST	LT	STa
1	W1 (6)	ND^a^	ND	7.3±1.6^A^	+^f^	+^k^
	W2 (11)	+^b^	+d,q	7.5±1.2^A^	+^g^	+^l^
2	W5 (14)	+^c^	ND	6.3±1.8^AB^	+^h^	+^m^
	W6 (8)	ND	+e,q	6.0±1.7^AB^	ND	+^n^
3	C1 (7)	5.6±1.3	ND	5.6±1.0^B^	+^i^	+^o^
	C2 (8)	5.2±1.1	ND	5.6±1.7^B^	+^j^	+^p^

a ND not detected,

b (n = 1) 3.7±0.1 log gene copy numbers g^−1^,

c (n = 3) 2.5±0.2–3.2±0.4 log gene copy numbers g^−1^,

d (n = 2) 6.5±0.1–10.5±0 log gene copy numbers g^−1^,

e (n = 1) 10.1±0 log gene copy numbers g^−1^,

f (n = 1) 4.7±0.2 log gene copy numbers g^−1^,

g (n = 1) 4.7±0.3 log gene copy numbers g^−1^,

h (n = 4) 4.7±0.1 log gene copy numbers g^−1^,

i (n = 1) 4.7±0.1 log gene copy numbers g^−1^,

j (n = 1) 4.2±0 log gene copy numbers g^−1^,

k (n = 1) 5.2±0 log gene copy numbers g^−1^,

l (n = 2) 4.2±0.1 log gene copy numbers g^−1^,

m (n = 5) 4.2±0.4–8.1±0 log gene copy numbers g^−1^,

n (n = 1) 4.4±0 log gene copy numbers g^−1^,

o (n = 1) 4.7±0.2 log gene copy numbers g^−1^,

p (n = 1) 3.8±0 log gene.

### Impact of diet and environment on fecal microbiota and enteric pathogen load

**Figure 3:**
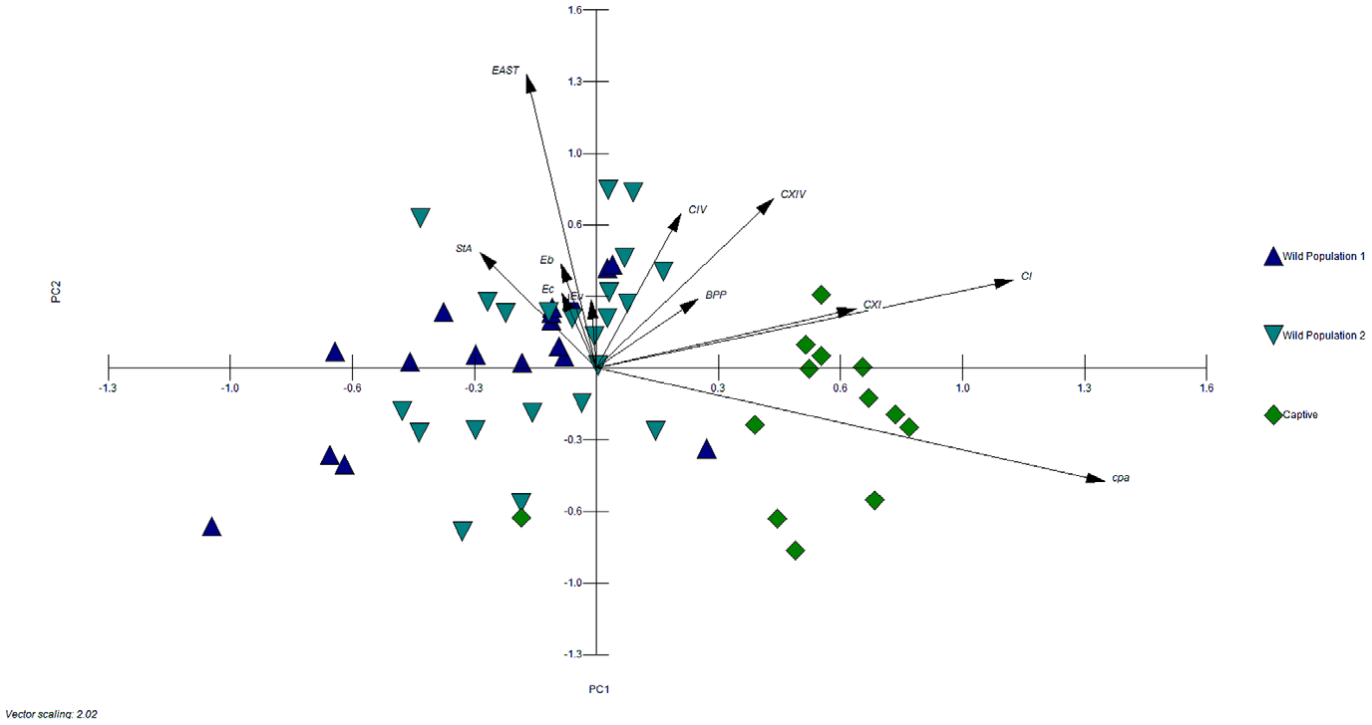
Correlation of housing conditions and stability of fecal bacterial communities in grizzly bears. Score and loading biplot indicating the correlation between habitat, fecal microbiota, diet, and enteric pathogens of individual wild (W1, W2, W5, and W6) and captive grizzly bears (C1, C2). The first two principal components PC1 (x-axis) and PC2 (y-axis) account for 43% of the total variance. Variables with low weights are not displayed. The three populations are indicated by colours and symbols (population 1, blue triangle; population 2, turquoise inverse triangle; population, 3 green square). Eu, total Eubacteria; Eb, *Enterobacteriaceae*; Ec, Enterococci; BPP, *Bacteroides-Prevotella-Porphyrmonas*; CI, *Clostridium cluster* I; CIV, *Clostridium* cluster IV; CXI, *Clostridium cluster* XI; CXIV, *Clostridium* cluster XIV; EAST, heat-stable enterotoxin; STa, shiga-like toxin A gene; cpa, *C. perfringens* alpha toxin gene A.

Principal component analysis was performed to identify possible correlations between fecal microbiota composition, diet and the presence of enteric pathogens. Wild and captive animals clustered separately along PC1, and no distinctive population dependent cluster was observable within the two wild populations (Figure 3). PC1 mostly explained the variables *Clostridium* clusters I and XI, *cpa* and the meat component of the diet (short arrow, data not shown). The small angle between loading variables *Clostridium* clusters I and XI indicated their positive correlation. In contrast, the variables Eubacteria, *Enterobacteriaceae*, enterococci correlated to PC2 together with the *E. coli* virulence factors.

## Discussion

Alberta grizzly bears inhabit areas that are being increasingly affected by the expansion of industrial activities and increases in human access to previously remote areas [14]. Human-caused habitat degradation and fragmentation exerts pressure on grizzly populations in Alberta. Low reproductive rates and specific habitat and food requirements during critical times of the year (e.g. reproductive period and pre-denning), make grizzly bears susceptible to human interference and could contribute to a decrease in long-term viability of grizzly populations. Habitat alteration clearly has the potential to negatively impact bear populations but also could relate to the detrimental effects of pathogenic bacteria and physiological stressors caused by inadequate food intake. Diseases impact wildlife health and their management requires a conjunct interdisciplinary effort to ensure species conservation [15].

Previous investigations of grizzly bear gut microflora were based on single samples from often unidentified animals, and did not follow the same individual over a period of time [6]. In contrast, the present study used GPS radiocollar technology to intensively monitor bears sampled randomly from the threatened population in Alberta.

The composition of fecal microbiota fluctuated strongly in individual animals especially for wild populations. Fluctuations of the intestinal microbiota of *Ursids* are attributable to the simple gut physiology and fast digestion times, and are likely enhanced by fluctuations of the diet of wild animals [2], [3], [5]. Confirming earlier studies, total bacterial counts in wild and captive grizzly bear feces were dominated by the facultative anaerobes *Enterobacteriacae* and enterococci [6]. *Clostridium* clusters I and XI outnumbered the *Bacteroides-Prevotella-Porphyrmonas* group as well as the *Clostridium* clusters IV and XIV in feces of captive animals. In wild animals, the abundance of *Clostridium* clusters I and XI was comparable to the *Bacteroides-Prevotella-Porphyrmonas* group. Members of the *Bacteroides-Prevotella-Porphyrmonas* and *Clostridium* clusters IV and XIVa are highly adapted to the utilization of plant polysaccharides and are the predominant populations in herbivorous animals. In omnivores such as humans, a high fibre diet results in enhanced abundance of *Bacteroidetes* [16]. In contrast, carnivores have high prevalence of *Clostridium* clusters I and XI, which include saccharolytic fibre-fermenting species, but also proteolytic or toxinogenic clostridia [17]. The majority of *Clostridiales* isolates from a clonal library of dogs was assigned to *Clostridium* cluster XI [18]; a protein rich diet increased the presence of *Clostridium* cluster I in cats and dogs [19], [20]. In wild polar bears predominantly feeding on seals and fish [21], *Clostridium* clusters I and XI prevailed. In our study, *Clostridum* clusters I and XI were most abundant in captive grizzly bears with a high likelihood of co-occurrence (r = 0.78, p<0.001). These results suggest that within members of the Carnivora a positive correlation exists between abundances of *Clostridium* clusters I and XI and diet protein content.

The presence of the pathogenic *C. perfringens* of *Clostridum cluster* I was also positively correlated to protein intake and negatively correlated to diet fibre content in grizzly bears. Even though considered healthy, grizzly bears consuming a regular animal protein based diet were more prone to carry *C. perfringens* than the wild population relying on a plant based diet. In wild chimpanzees, extensive fibre consumption reduced the occurrence of *C. perfringens* compared to captive animals consuming lower amounts of fibre [22]. Strict carnivores such as polar bears, which are carriers of *C. perfringens* [23], [24] may not have the mechanism to regulate the prevalence of *C. perfringens* due to lack of fibre in their diet. Because certain strains of *C. perfringens* caused mortality in *Ursids* [25], implementation of nutrition plans incorporating higher proportions of plant components could benefit bears kept in captivity. Such plans and regular monitoring of fecal microbiota and pathogens may also benefit re-establishing bear populations originating from captive bread individuals [26], [27].

Interestingly, three samples were analyzed positive for the presence of *C. sordellii*, which in lions has been associated with sudden death, and which can trigger toxic shock syndrome in humans [28], [29].

In contrast to *C. perfringens*, no correlation of abundance of pathogenic *E. coli* (ETEC and EAEC), diet, or study area was observed. The relation between presence of *E. coli* virulence factors and disease occurrence is not always conclusive. However, the presence of pathogenic *E. coli* is connected to diarrhea in cats, dogs and young farm animals [30], [31].

This is the first longitudinal study monitoring the fecal microbiota of grizzly bears and we found that the composition of the fecal microbiota is affected by location, housing and diet. This study also shows that wild and captive grizzly bears carry pathogenic *C. perfringens* and *E. coli*, as well as *C. sordellii*. In humans, ecological changes of the ancient microbiota affect physiology and ultimately health [32]. In bears and other wild animals, habitat changes that modify the availability of natural foods may affect the variability of the fecal microbiota and increase susceptibility to pathogenic bacteria. Following the decade old suggestion to incorporate wildlife health assessments into conservation [33], this study provides a framework for carrying out longitudinal research on the intestinal microbiota and enteric pathogen load in wild animals that provides data beneficial to wildlife conservation.

## Materials and Methods

### Study areas of wild grizzly bear populations

The two bear populations inhabit south-western (population 1) and west-central (population 2) Alberta, Canada (Figure 1). Population 1 is located in an area characterized by a rapid transition from gently rolling agricultural land in the east to steep mountains in the west, with a gradually widening area of foothills beginning in the central portion of the study area (49°2′ N −113°54′ W). Upper elevation levels range from 1100 m in the north-eastern portion of the study area to over 2700 m in Waterton Lakes National Park. The eastern portion of the study area is largely agricultural, in the form of cattle ranching, and is a mosaic of trembling aspen (*Populus tremuloides*), balsam poplar (*Populus balsamifera*) and willow (*Salix* spp.) mixed with large patches of open pasture and cropland. Population 2 inhabits the eastern slopes of the Rocky Mountains and foothills at the eastern boundary of Jasper National Park (53°150′ N 118°300′ W). The vegetation within the sampling area consists of montane, conifer and sub-alpine forests, alpine meadows, and shrubs. The highest elevation is 3680 m, with rocky peaks, steep mountain sides and flatter, narrow valleys. Both regions receive moderate to high amounts of human disturbance from oil and gas development, logging and recreation. South-western Alberta has agricultural and ranching lands which are absent in west-central Alberta, but the latter has increased levels of open-pit mining activities as well as areas with minimal human disturbance in the more mountainous sections.

### Sampling from wild grizzly bears (populations 1 and 2)

During the April-November 2009 sampling period, 8 grizzly bears of the two populations were monitored as part of a larger study on grizzly bear foraging and movement ecology. Animals were equipped with GPS radiocollars with remote data upload capabilities (Televilt, Lindesberg, Sweden; University of Alberta Animal Care and Use Committee for Biosciences Protocols 552712 and 552812). Individual bears were monitored for varying periods due to timing of capture operations, collar failure, and removal of collars by the bears themselves. Throughout the study period, bears were located using fixed-wing aircraft, helicopter, or from the ground. GPS data were remotely uploaded from the collars without the need for bear recapture. A sample of sites at which collared individuals of populations 1 and 2 had spent a substantial amount of time were visited by field teams after the animals had left the area. Because grizzly bears and black bears are sympatric in both populations, only scat samples which were unambiguously attributed to grizzly bears were included in microbiological analysis. Samples were collected from areas in which radiocollared grizzly bears had spent several hours, most often from bedding sites as confirmed through GPS positioning, a bedding depression and the presence of grizzly bear hair in the actual bed. In contrast to previous studies on the fecal microbiota of wild hominids [34], the age of samples could be assigned in this study. The GPS collar technology allowed precise identification of individual grizzly bears that had used specific sites, along with information on date and time when the bear was there. For population 1, approximately 40 mL of feces were collected in sterile zilpoc bags. Microbiota analysis was conducted on a portion of the sample whereas visual identification of diet contents was conducted on the remaining portion of the same feces. In population 2, from each feces found in the field, two samples were collected and placed in separate 50 mL sterile plastic tubes. One tube was destined for microbiota analysis and the other was used for visual identification of diet contents. Samples were stored frozen until analysis.

### Sampling from captive grizzly bears (population 3)

Fecal samples from one male and one female grizzly bear housed at the Calgary Zoo were obtained in cooperation with the zoo staff at regular intervals from February to October 2009 (Biological Research Permit 2009-01). Only feces which could be assigned to the male or the female were used in this study. Samples were between 0 and 24 h old at collection and were frozen immediately until analysis. The animals were fed their regular diet consisting of dog kibble (male 26%, female 23%; dog kibble contains chicken and corn), fish (male 37%, female 26%) and fruit and vegetables (male 37%, female 51%). Feed was freely accessible.

### Visual diet analysis of wild grizzly bears

Thirty mL of each sample were autoclaved and rinsed through a 0.5 mm metal sieve to remove small soil and sand particles that the bears had ingested together with food. Samples were dried in a fume hood and transferred to wide diameter petri dishes. Using tweezers and a dissecting microscope, the fecal sample was spread over the dish, grouping similar items together. All items were laid flat on a petri dish with a grid of 2×2 cm squares placed below. The grid was used to determine the percentage of each food item in the fecal sample. This procedure provided information on the proportion of various food items in bear feces for the same standardized volume of feces. Fecal content of wild grizzly bears was grouped into four categories: plants (forbs, grasses, cultivated cereals, roots, stems, leaves and fruits), mammals (hair, bone, meat), insects, and miscellaneous (soil, rock, wood).

### DNA isolation and quantitative PCR (qPCR) for determination of fecal microbiota and enteric pathogen load

DNA was isolated from feces with Qiamp DNA stool mini kit (Qiagen, Mississauga, Canada) according to instructions of the manufacturer. This kit ensures high lysis efficiency and has a proven high consistency in DNA isolation from ecological samples [35]. Duplicate test extractions from the same sample and consequent qPCR amplification of some of the bacterial target groups showed a variation in gene copy numbers to a maximum of 8%. For three samples collected during bear capture, fecal bacterial DNA was isolated and samples were analyzed as controls to identify the range of bacterial populations in wild grizzly bear feces (Table S2). Fecal bacterial composition was analyzed by qPCR using group specific primers targeting the 16S rRNA gene (Table S3). Only samples of population 1 and 2, which were between 6–16 and 15–24 days old at collection, respectively, were included in bacterial population analysis.

To generate standard curves, DNA was isolated of representative strains of the bacterial groups analysed and from strains of *C. perfringens* (*C. perfringens* alpha-toxin gene *cpA* positive), *C. botulinum* Group I (*C. botulinum* neurotoxins A and B), *C. difficile* (toxin B gene *tcdB*), all obtained from the culture collection of the Food Microbiology Laboratory, University of Alberta, and of *E. coli* ECL13086 (O149, virotype STa∶STb∶LT∶EAST1∶F4), purchased from *Escherichia coli* Laboratory, University of Montréal, Canada. The *C. sordelli* phospholipase C was amplified from fecal samples, purified and sequenced (MacrogenUSA, Rockville, USA) to verify its identity. The amplicon was 97% identical (in 154 AA) to a phospholipase C *csp*C of *C. sordellii* ATCC9714 (AB061868). Standard curves were generated from PCR amplicons according to Metzler-Zebeli et al. [8]. The *C. botulinum* neurotoxin A gene was qualitatively analysed only. For SybrGreen based reactions, master mixes (25 µL) contained 12.5 µL QuantiFast SYBR green master mix (Qiagen, Mississauga, ON, Canada), 1 µL DNA and 0.05 pmol L^−1^ primer. Melting curve analysis and size determination of amplificates on agarose verified amplification of the targeted fragments. The master mixes (25 µL) of PCR reactions using Taqman probes contained 12.5 µL Taqman Fast (Applied Biosystems), 1 µL DNA, 0.05 pmol L^−1^ primer, and 1 µmol L^−1^ probe. The PCR cycle was set to 94°C, 5 min initial denaturation followed by 40 cycles 94°C 15 s, 15 s annealing (temperatures indicated in Table S3) and extension at 72°C for 30 s.

### Statistical analysis

Differences of bacterial log gene copy numbers between populations were analyzed using one-way ANOVA (SigmaPlot 11, Systat Software). If data was not distributed normally, Kruskal-Wallis One Way Analysis of Variance on Ranks was applied. Results are shown graphically in Figure 2. Populations that do not share a common superscript (A,B) are significantly different (p<0.05). Abundance of *Clostridium* clusters I and XI was also compared in wild and captive animals (Kruskal-Wallis One Way Analysis of Variance on Ranks), significant differences (p<0.05) are indicated by small letters (a,b). To investigate correlations between bacterial groups within a population, Spearman rank order correlation was performed using (SigmaPlot 11). Multi-variate Statistical Package (MVSP) was used for principal component analysis (PCA) [36]. PCA was carried out using bacterial group gene copies, virulence genes and diet components as variables. Only bears from which at least six samples were obtained were included in statistical analysis.

## Supporting Information

Figure S1 **Wild grizzly bear diet content.** Diet content in the feces of a bears W1 (A) and W2 (B) of population 1, and of bears W5 (C) and W6 (D) of population 2. Diet content was categorized as plant material (plant), mammalian matter (mammal), insect and miscellaneous (misc).
(TIF)

Table S1 Bacterial populations [log DNA gene copy numbers g^−1^ feces] in the feces of wild and captive grizzly bears. Average values were calculated from samples of individual bears and were analysed using one-way ANOVA.
(DOCX)

Table S2 Selected bacterial groups present in the feces of three adult wild grizzly bears and a grizzly bear cub at bear capture. Samples were analyzed as a control to identify the range of bacterial populations in wild grizzly bear feces. Bear W5 was captured early in the season. As counts of bacterial groups were within ranges also observed in collected samples, we consider results obtained in this study valuable. The fecal microbiota of the grizzly bear cub was distinctively different with the major proportion being represented by *Enterobacteriaceae*.
(DOCX)

Table S3 Primers used in this study.
(DOCX)
