# Supplementary material for: Diet and Environment Shape Fecal Bacterial Microbiota Composition and Enteric Pathogen Load of Grizzly Bears
Source: PLoS One. 2011 Dec 15;6(12):e27905. doi: 10.1371/journal.pone.0027905 (PMC3240615; doi:10.1371/journal.pone.0027905)
Supplement: Table S1 — Bacterial populations [log DNA gene copy numbers g−1 feces] in the feces of wild and captive grizzly bears. Average values were calculated from samples of individual bears and were analysed using one-way ANOVA. (DOCX) [file pone.0027905.s002.docx]

**Table S1** Bacterial populations [log DNA gene copy numbers g^-1^ feces] in the feces of wild and captive grizzly bears. Average values were calculated from individual samples and analysed using one-way ANOVA.

| Population | Animal (number of samples) | **Total Eu-bacteria** | | | ***Entero-bacteriacae*** | | **Entero-cocci** | | **LPLW^a^** | | **BPP^b^** | | **CI^c^** | **CXI^d^** | **CXIV^e^** | |
| --- | --- | --- | --- | --- | --- | --- | --- | --- | --- | --- | --- | --- | --- | --- | --- | --- |
| 1 | W1 (6)^f^ | 9.3±0.4^A^ | | | 8.4±0.9^A^ | | 7.3±0.9^AB^ | | 5.3±0.6^A^ | | 6.1±1.7^A^ | | 6.2±0.7^A^ | 6.9±0.5^A^ | 3.2±1.2^A^ | |
|  | W2 (11) ^f^ | 9.2±0.8^A^ | | | 8.2±1.2^A^ | | 7.4±1.2^AB^ | | 5.3±0.6^A^ | | 5.8±0.9^A^ | | 6.3±0.8^A^ | 6.7±0.6^A^ | 4.3±0.8^A^ | |
|  | W3 (2) | 8.9±0 | | | 8.5±0.4 | | 8.2±0.8 | | 5.6±0.1 | | 5.8±0.2 | | 4.8±0.2 | nd^g^ | 3.3±0 | |
|  | W4 (4) | 9.3±0.6 | | | 7.2±0.7 | | 7.8±0.7 | | 5.0±1.6 | | 5.2±0.9 | | 4.1±0 | nd | 3.7±1.6 | |
| 2 | W5 (14) ^f^ | 9.2±0.5^A^ | | | 8.2±0.8^A^ | | 7.9±1.1^AB^ | | 5.8±0.5^A^ | | 5.7±1.7^A^ | | 5.3±1.1^B^ | 6.4±0.5^A^ | 4.1±1.9^A^ | |
|  | W6 (8)^f^ | 9.3±0.5^A^ | | | 7.8±0.7^A^ | | 7.7±0.5^B^ | | 5.7±0.5^A^ | | 6.7±1.3^A^ | | 5.9±1.1^B^ | 6.2±2.3^A^ | 4.7±1.6^A^ | |
|  | W7 (3) | 9.4±0.6 | | | 8.0±1.4 | | 7.7±0.7 | | 5.9±0.3 | | 6.9±1.3 | | 5.1±0.4 | nd | 4.8±2.1 | |
|  | W8 (3) | 9.2±0.9 | | | 6.9±0.7 | | 7.0±0.4 | | 5.4±1.1 | | 6.2±1.6 | | 4.8±0.4 | nd | 3.8±0.2 | |
| 3 | C1 (7) ^f^ | 8.9±0.5^B^ | | | 7.9±0.9^A^ | | 7.3±0.8^A^ | | 6.0±0.6^A^ | | 5.9±0.7^A^ | | 7.2±0.7^A^ | 7.8±1.0^B^ | 4.3±1.4^A^ | |
|  | C2 (8) ^f^ | | 8.8±0.6^B^ | 7.5±1.3^A^ | | 7.1±0.5^A^ | | 5.8±0.9^A^ | | 6.3±0.7^A^ | | 6.8±0.8^A^ | | 7.9±0.5^B^ | | 4.9±1.2^A^ |

^a^*Lactobacillus, Pediococcus,* *Leuconostoc, Weissella* spp., ^b^*Bacteroides-Prevotella-Porphyrmonas*, ^c^*Clostridium* cluster I, ^d^*Clostridium* cluster XI, ^e^*Clostridium* cluster XIV, ^f^animals included in statistical analysis, ^g^not determined. ^A,B^Populations that do not share a common superscript (A,B) are significantly different (p<0.05).
