# Supplementary material for: Diet and Environment Shape Fecal Bacterial Microbiota Composition and Enteric Pathogen Load of Grizzly Bears
Source: PLoS One. 2011 Dec 15;6(12):e27905. doi: 10.1371/journal.pone.0027905 (PMC3240615; doi:10.1371/journal.pone.0027905)
Supplement: Table S2 — Selected bacterial groups present in the feces of three adult wild grizzly bears and a grizzly bear cub at bear capture. Samples were analyzed as a control to identify the range of bacterial populations in wild grizzly bear feces. Bear W5 was captured early in the season. As counts of bacterial groups were within ranges also observed in collected samples, we consider results obtained in this study valuable. The fecal microbiota of the grizzly bear cub was distinctively different with the major proportion being represented by Enterobacteriaceae. (DOCX) [file pone.0027905.s003.docx]

**Table S2** Selected bacterial groups present in the feces of three adult wild grizzly bears and a grizzly bear cub at bear capture. Samples were analyzed as a control to identify the range of bacterial populations in wild grizzly bear feces. Bear W5 was captured early in the season. As counts of bacterial groups were within ranges also observed in collected samples, we consider results obtained in this study valuable. The fecal microbiota of the grizzly bear cub was distinctively different with the major proportion being represented by *Enterobacteriaceae.*

| Animal | **Total Eubacteria** | ***Entero-bacteriaceae*** | **Entero-cocci** | **LPLW^a^** | **BPP^b^** | **CI^c^** | | **CXIV^d^** |
| --- | --- | --- | --- | --- | --- | --- | --- | --- |
| W5 | 8.7±0 | 8.7±0.1 | 5.8±0 | 5.7±0.1 | 9.4±0.2 | 6.4±0.1 | | 7.8±0.1 |
| W8 | 8.5±0.1 | 6.4±0.1 | 6.7±0.1 | 6.1±0.3 | 5.1±0 | 4.4±0.2 | | 3.7±0.1 |
| G117^e^ | 8.1±0 | 7.9±0.1 | 7.8±0 | ND | 4.4±0 | 5.1±0.1 | | 2.2±0 |
| Cub | 9.0±0.1 | 9.4±0.1 | 6.5±0.1 | 4.1±0.2 | 4.8±0 | nd^f^ | nd | |

^a^*Lactobacillus, Pediococcus,* *Leuconostoc, Weissella* spp., ^b^*Bacteroides-Prevotella-Porphyrmonas*, ^c^*Clostridium* cluster I, ^d^*Clostridium* cluster CXIV, ^e^no further samples were obtained from this bear, ^f^not determined
