# Supplementary material for: Diet and Environment Shape Fecal Bacterial Microbiota Composition and Enteric Pathogen Load of Grizzly Bears
Source: PLoS One. 2011 Dec 15;6(12):e27905. doi: 10.1371/journal.pone.0027905 (PMC3240615; doi:10.1371/journal.pone.0027905)
Supplement: Table S3 — Primers used in this study. (DOCX) [file pone.0027905.s004.docx]

**Table** **S3** Primers used in this study.

| Target |  | **Primer** | **5’-3’** | **Annealing temperature [ºC]** | **Reference** |
| --- | --- | --- | --- | --- | --- |
| Bacterial groups | Total Eubacteria | For | CGGYCCAGACTCCTACGGG | 60 | 0 |
|  |  | Rev | TTACCGCGGCTGCTGGCAC |  |  |
|  | *Enterobacteriacae* | For | GTTAATACCTTTGCTCATTGA | 60 | 2 |
|  |  | Rev | ACCAGGGTATCTAATCCTGTT |  |  |
|  | *Enterococcus* | For | CCCTTATTGTTAGTTGCCATCATT | 60 | 3 |
|  |  | Rev | ACTCGTTGTACTTCCCATTGT |  |  |
|  | *Lactobacillus*, *Pediococcus*, *Leuconostoc, Weissella* spp. | For | AGCAGTAGGGAATCTTCCA | 60 | 4 |
|  |  | Rev | CACCGCTACACATGGAG |  | 5 |
|  | *Bacteroides-Prevotella-Porphyrmonas* group | For | GGTGTCGGCTTAAGTGCCAT | 60 | 3 |
|  |  | Rev | CGGAYGTAAGGGCCGTGC |  |  |
|  | *Clostridium* cluster I | For | ATGCAAGTCGAGCGAKG | 60 | 3 |
|  |  | Rev | TATGCGGTATTAATCTYCCTTT |  |  |
|  | *Clostridium* cluster IV (*C. leptum* -*Faecalibacterium prausnitzii* subgroup) | For | GCACAAGCAGTGGAGT | 60 | 6 |
|  |  | *Rev* | *CTTCCTCCGTTTTGTCAA* |  |  |
|  | *Clostridium* cluster XI | For | ACGCTACTTGAGGAGGA | 60 | 7 |
|  |  | Rev | GAGCCGTAGCCTTTCACT |  |  |
|  | *Clostridium* cluster XIVa and XIVb (*C. coccoides* - *Eubacterium rectale* subgroup) | For | AAATGACGGTACCTGACTAA | 60 | 8 |
|  |  | Rev | CTTTGAGTTTCATTCTTGCGAA |  |  |
|  | *Bifidobacterium* spp. | For | TCGCGTCYGGTGTGAAAG | 60 | 3 |
|  |  | Rev | CCACATCCAGCRTCCAC |  |  |
| *C. perfringens* alpha toxin A gene | CRPF *cpA* | For | GCTAATGTTACTGCCGTTGA | 55 | 9 |
|  |  | Rev | CCT CATTAGTTTTGCAACC |  |  |
|  |  | Probe | FAM-GCGCAGGACATGTTAAGTTTG-TAMRA |  |  |
| *C. difficile* toxin B gene | CPRF *tcdB* | For | GAAAGTCCAAGTTTACGCTCAAT | 55 | 10 |
|  |  | Rev | GCTGCACCTAAACTTACACCA |  |  |
|  |  | Probe | FAM-ACAGATGCAGCCAAAGTTGTTGAATT-TAMRA |  |  |
| *C. botulinum* toxin genes | *C. botulinum* toxin A gene | For | GTGATACAACCAGATGGTAGTTATAG | 55 | 11 |
|  |  | Rev | AAAAAACAAGTCCCAATTATTAACTTT |  |  |
|  | *C. botulinum* toxin B gene | For | GGAGAAGTGGAGCGAAAAA | 55 | 12 |
|  |  | Rev | TTCCCTTGATGCAAAATGAT |  |  |
|  |  | Probe | CCTGGGCCAGTTTTAAATGA |  |  |
| *C. sordellii* phospholipase C | *csp*C | For | TAAAGATGCAGTACCTAATAAGGATTT | 55 | 13 |
|  |  | Rev | TTCCTGAAATTTGATCTTCTGAAACC |  |  |
| *Enterobacteriaceae* toxin genes | LT | For | CTATTACAGAACTATGTTCGG | 56 | 14 |
|  |  | Rev | TACTGATTGCCGCAATTG |  |  |
|  | EAST | For | TGCCATCAACACAGTATATCC | 56 | 14 |
|  |  | Rev | GCGAGTGACGGCTTTGT |  |  |
|  | STa | For | ATGAAAAAGCTAATGTTGGC | 56 | 14 |
|  |  | Rev | TACAACAAAGTTCACAGCAG |  |  |
|  | STb | For | AATATCGCATTTCTTCTTGC | 56 | 14 |
|  |  | Rev | GCATCCTTTTGCTGCAAC |  |  |

**References**

1. Lee D-H, Zo Y-G, Kim S-J (1996) Nonradioactive method to study genetic profiles of natural bacterial communities by PCR-single-strand-conformation polymorphism. Appl Environm Microbiol 62: 3112-3120.
2. Malinen E, Kassinen A, Rinttilä T, Palva A (2002) Comparison of real-time PCR with SYBR Green I or 59-nuclease assays and dot-blot hybridization with rDNA targeted oligonucleotide probes in quantification of selected faecal bacteria. Microbiol 149: 269-277.
3. Rinttilä T, Kassinen A, Malinen E, Krogius L, Palva A (2004). Development of an extensive set of 16S rDNA-targeted primers for quantification of pathogenic and indigenous bacteria in fecal samples by real-time PCR. J Appl Microbiol 97: 1166-1177.
4. Heilig HGHJ, Zoetendal EG, Vaughan EE, Marteau P, Akkermans ADL et al. (2002) Molecular diversity of *Lactobacillus* spp. and other lactic acid bacteria in the human intestine as determined by specific amplification of 16S ribosomal DNA. Appl Environm Microbiol 68: 114-123.
5. Walter J, Hertel C, Tannock GW, Los CM, Munro K et al. (2001) Detection of *Lactobacillus, Pediococcus, Leuconostoc,* and *Weissella* species in human feces by using group-specific PCR primers and denaturing gradient gel electrophoresis. Appl Environm Microbiol 67: 2578-2585.
6. Matsuki T, Watanabe K, Fujimoto J, Takada T, Tanaka R (2004) Use of 16S rRNA gene-targeted group specific primers for real-time PCR analysis of predominant bacteria in human feces. Appl Environm Microbiol 70: 7220-7228.
7. Song Y, Liu C, Finegold SM (2004) Real-time PCR quantitation of clostridia in feces of autistic children. Appl Environm Microbiol 70: 6459–6465.
8. Matsuki T, Watanabe K, Fujimoto J, Miyamoto Y, Takada T et al. (2002) Development of 16S rRNA gene-targeted group specific primers for the detection and identification of predominant bacteria in human feces. Appl Environm Microbiol 68: 5445-5451.
9. Messelhäusser U, Zucker R, Elmer-Englhard D, Busch U, Hörmansdorfer S et al. (2007a). Nachweis und Charakterisierung von *Clostridium perfringens* mittels real-time-PCR. J Verbrauch Lebensm 2: 194–197.
10. Van den Berg RJ, Kuijper EJ, Bruijnsteijn van Coppenraet LES, Claas ECJ (2006) Rapid diagnosis of toxinogenic *Clostridium difficile* in faecal samples with internally controlled real-time PCR. Clin Microbiol Infect 12: 184-186.
11. FDA Bacteriological Analytical Manual (2011) Chapter 17 *Clostridium botulinum*. Available: http://www.fda.gov/Food/ScienceResearch/LaboratoryMethods/BacteriologicalAnalyticalManualBAM/ucm070879.htm. Accessed May 2011.
12. Messelhäusser U, Zucker R, Ziegler D, Elmer-Englhard D, Kleih W et al. (2007b) Nachweis von *Clostridium botulinum* Typ A, B, E und F mittles real-time-PCR. J Verbrauch Lebensm 2: 198-201.
13. Fischer M, Bhatnagar J, Guarner J, Reagan S, Hacker JK et al. (2005) Fatal toxic shock syndrome associated with *Clostridium sordellii* after medical abortion. New Engl J Med 353: 2352-2360.
14. Han W, Liu B, Cao B, Beutin L, Krüger U et al. (2007) DNA microarray-based identification of serogroups and virulence gene patterns of *Escherichia coli* isolates associated with porcine postweaning diarrhea and edema disease. Appl Environm Microbiol 73:4082–4088.
